# Disparities and Trends in Birth Outcomes, Perinatal and Infant Mortality in Aboriginal vs. Non-Aboriginal Populations: A Population-Based Study in Quebec, Canada 1996–2010

**DOI:** 10.1371/journal.pone.0138562

**Published:** 2015-09-23

**Authors:** Lu Chen, Lin Xiao, Nathalie Auger, Jill Torrie, Nancy Gros-Louis McHugh, Hamado Zoungrana, Zhong-Cheng Luo

**Affiliations:** 1 Ministry of Education-Shanghai Key Laboratory of Children's Environmental Health, Xinhua Hospital, Shanghai Jiao-Tong University School of Medicine, Shanghai, 200092, China; 2 Department of Obstetrics and Gynecology, Sainte-Justine Hospital Research Center, University of Montreal, 3175 Cote-Sainte-Catherine, Montreal, Quebec, H3T 1C5, Canada; 3 University of Montreal Hospital Research Centre, Montreal, Quebec, Canada; 4 Cree Board of Health and Social Services of James Bay, Mistissini, Quebec, G0W 1C0, Canada; 5 First Nations of Quebec and Labrador Health and Social Service Commission, Wendake, Quebec, G0A 4V0, Canada; 6 Nunavik Regional Board of Health and Social Services, Kuujjuaq, Quebec, J0M1C0, Canada; 7 Institut National de Santé Publique du Québec, Montreal, Quebec, H2P 1E2, Canada; University of Cincinnati, UNITED STATES

## Abstract

**Background:**

Aboriginal populations are at substantially higher risks of adverse birth outcomes, perinatal and infant mortality than their non-Aboriginal counterparts even in developed countries including Australia, U.S. and Canada. There is a lack of data on recent trends in Canada.

**Methods:**

We conducted a population-based retrospective cohort study (n = 254,410) using the linked vital events registry databases for singleton births in Quebec 1996–2010. Aboriginal (First Nations, Inuit) births were identified by mother tongue, place of residence and Indian Registration System membership. Outcomes included preterm birth, small-for-gestational-age, large-for-gestational-age, low birth weight, high birth weight, stillbirth, neonatal death, postneonatal death, perinatal death and infant death.

**Results:**

Perinatal and infant mortality rates were 1.47 and 1.80 times higher in First Nations (10.1 and 7.3 per 1000, respectively), and 2.37 and 4.46 times higher in Inuit (16.3 and 18.1 per 1000, respectively) relative to non-Aboriginal (6.9 and 4.1 per 1000, respectively) births (all p<0.001). Compared to non-Aboriginal births, preterm birth rates were persistently (1.7–1.8 times) higher in Inuit, large-for-gestational-age birth rates were persistently (2.7–3.0 times) higher in First Nations births over the study period. Between 1996–2000 and 2006–2010, as compared to non-Aboriginal infants, the relative risk disparities increased for infant mortality (from 4.10 to 5.19 times) in Inuit, and for postneonatal mortality in Inuit (from 6.97 to 12.33 times) or First Nations (from 3.76 to 4.25 times) infants. Adjusting for maternal characteristics (age, marital status, parity, education and rural vs. urban residence) attenuated the risk differences, but significantly elevated risks remained in both Inuit and First Nations births for the risks of perinatal mortality (1.70 and 1.28 times, respectively), infant mortality (3.66 and 1.47 times, respectively) and postneonatal mortality (6.01 and 2.28 times, respectively) in Inuit and First Nations infants (all p<0.001).

**Conclusions:**

Aboriginal vs. non-Aboriginal disparities in adverse birth outcomes, perinatal and infant mortality are persistent or worsening over the recent decade in Quebec, strongly suggesting the needs for interventions to improve perinatal and infant health in Aboriginal populations, and for monitoring the trends in other regions in Canada.

## Introduction

Aboriginal populations are at higher risks of adverse birth outcomes (e.g. preterm birth), perinatal and infant mortality than their non-Aboriginal counterparts in many countries including Australia, the U.S., and Canada [1–14]. Such glaring health inequalities at the beginning of life are partly attributable to disadvantaged socioeconomic conditions in Aboriginal populations [7–9]. Timely and reliable data on trends in birth outcomes, perinatal and infant mortality are essential to develop appropriate policies, programs and interventions for narrowing the disparities among Aboriginal vs. non-Aboriginal populations. However, there is a lack of data on recent trends in birth outcomes, perinatal and infant mortality for Aboriginal populations in Canada.

There are three groups of Aboriginal peoples in Canada: First Nations (North American Indians), Inuit and Metis. In most Canadian provinces, there is a lack or poor-quality of data on Aboriginal birth outcomes, perinatal and infant mortality due to the lack or poor-quality of Aboriginal birth identifiers on birth registrations [15]. There remains a lack of national data on birth outcomes and infant mortality for Aboriginal populations in Canada due to data deficiencies and jurisdictional barriers in the Canadian multi-jurisdictional vital statistics systems [15,16]. In Quebec, previous studies were mostly based on mother tongue to identify Aboriginal (First Nations, Inuit) births. However, about half of First Nations no longer speak an Aboriginal mother tongue, according to the Canada 2006 census. The present study sought to determine recent trends in birth outcomes, perinatal and infant mortality disparities comparing First Nations, Inuit vs. non-Aboriginal populations in Quebec, using multiple sources of information to identify Aboriginal births. We did not study Metis birth outcomes because Metis births in Quebec could not be identified in any available birth-related administrative health databases.

## Methods

### Study design and population

This was a population-based retrospective birth cohort study, based on the linked vital events registry databases in Quebec, Canada. In Quebec, all vital events (stillbirths at 500+ grams, all live births, all deaths) are required to be registered by law using standard registration forms, and are routinely collected through the Ministère de la Santé et des Services Sociaux (MSSS) registry system. Vital events are reviewed and captured into electronic databases at the Institut de la Statistique du Québec (ISQ). The study birth cohort was constituted based on all singleton births to Quebec residents in 1996–2010, the most recent databases available at the time of the approval of the project by the Commission for Access to Information (CAI) in Quebec in 2013. The initial research protocol proposed to include all singleton births (Aboriginal and non-Aboriginal) in Quebec 1996–2010 in the study cohort. However, the CAI granted the inclusion of all identified *Aboriginal* singleton births, but did not permit the inclusion of all *non-Aboriginal* singleton births and recommended a 20% random sample of non-Aboriginal births for inclusion in the study cohort since the total number of non-Aboriginal singleton births was huge (about 1.15 million). The representativeness of the 20% sampled non-Aboriginal singleton birth cohort was excellent: identical rates of preterm birth, low birth weight, stillbirth and infant death, and identical mean maternal age and year of education were observed in the sampled singleton non-Aboriginal birth cohort vs. the total singleton non-Aboriginal birth cohort. In respecting the ISQ data reporting confidentiality rules, birth numbers are reported to the nearest 10. The final study birth cohort included 20,190 First Nations, 4,260 Inuit and 229,960 non-Aboriginal singleton births in Quebec, 1996–2010.

### Ethics statement

The study was approved by the Research Ethics Board of Sainte-Justine hospital research center. Informed consent was waivered because the study was based on administrative health databases. Patient records/information was anonymized and de-identified prior to data analysis. Approvals of the study were also obtained from the ISQ, the CAI, the Commission for Access to Information of the Aboriginal Affairs and Northern Development Canada, and three major Aboriginal community health organizations in Quebec—the Cree Board of Health and Social Services of James Bay, the First Nations of Quebec and Labrador Health and Social Service Commission, and the Nunavik Regional Board of Health and Social Services.

### Linkage of live births to infant death records

Linkage of live births (1996–2010) to infant death records (1996–2011) were based on infant’s sex, first name, last name, date of birth and birth weight, residential municipality and postal code, and mother’s/father’s first name, last name, mother’s residential municipality and postal code. The record linkage used probabilistic linkage technique, and the validity of the methodology has been well documented in Canadian vital events data linkage [17]. External validity of the infant death data linkage was excellent as indicated by comparisons of infant mortality rates by mother tongue group (First Nations, Inuit and Non-Aboriginal) for singleton births in Quebec 2001–2005 using Statistics Canada’s linked birth and infant death database vs our Quebec’s linked birth and infant death database: virtually identical infant mortality rates were observed (absolute rate differences within 0.1 per 1000).

### Identification of Aboriginal births

Aboriginal births were identified using three sources of information: 1) mother tongue–a birth was considered a First Nation or Inuit if the mother or father reported a First Nation or Inuit mother tongue on the birth registration, respectively; 2) residential postal code and municipality name–a birth was considered a First Nation if the mother’s residential postal code and/or municipality name corresponded to a First Nation community/reserve (about 90% of residents are First Nations, according to the Canada 2006 census) in Quebec, while a birth was considered an Inuit if the mother’s residential postal code / municipality name corresponded to an Inuit village / municipality in Nunavik (about 93% of residents are Inuit, according to the Canada 2006 census); 3) Indian Registration System (IRS) membership–a birth was considered a First Nation if either the mother or father was identified as an IRS member, according to the IRS membership database for all members (including deceased) up to December 2012 in Canada. The IRS membership database was provided by Aboriginal Affairs and Northern Development Canada which routinely collects and updates the IRS membership database every year. The linkage of births to IRS records was based on first name, last name, sex and date of birth of the infant, mother and father.

We created a composite Aboriginal birth identifier using all the three sources of information. A birth was considered a First Nation if the birth was identified as a First Nation by anyone of the three sources of information (mother tongue, postal code / municipality name, IRS membership). There were 7,020 singleton births identified as First Nations by mother tongue, 16,150 by residential postal code / municipality name, and 17,790 by IRS membership, respectively, while the total number of First Nations singleton births identified by anyone of the three methods was 20,190 (due to substantial overlaps in identified First Nations births by different methods). This total birth number is close to the estimated total number of births (n = 20700) based on self-reported First Nations children under 5 years of age in Quebec according to the 2006 Census. A birth was considered an Inuit if the birth was identified as an Inuit by mother tongue or postal code. There were 3,560 singleton births identified as Inuit by mother tongue, 4,190 singleton births identified as Inuit by residential postal code / municipality name, while the total number of Inuit singleton births identified by either method was 4,260. Exploratory analyses were conducted to examine Aboriginal birth outcomes by different Aboriginal birth identifiers.

Too few stillbirths and infant deaths could be identified through the linkage to the IRS membership database. Therefore, the IRS identifier was used in the analyses on birth outcomes, but was not used in the analyses on perinatal and infant mortality in First Nations.

### Rural vs urban residence

Based on geocoding postal code and municipality name [18] of the mother’s place of residence at the time of birth registration for her baby, rural and urban areas were defined according to Statistics Canada’s recommended definition: all census metropolitan areas and census agglomeration areas with > = 10,000 people were defined as urban, the residual areas are rural [19].

### Outcomes

Outcomes included preterm birth (<37 completed weeks of gestation), small-for-gestational-age (SGA, birth weight <10^th^ percentile, based on the Canadian fetal growth standards [20]), large-for-gestational-age (LGA, >90^th^ percentile), low birth weight (<2500 g), high birth weight (>4000 g), stillbirth (fetal death ≥20 weeks and ≥500 g), neonatal (0–27 days after birth) death, postneonatal (28–364 days after birth) death, perinatal death (stillbirths plus neonatal deaths) and infant death (neonatal deaths plus postneonatal deaths).

Causes of infant death were categorized according to the classification of the International Collaborative Effort on Perinatal and Infant Mortality[21], based on International Classification of Diseases (ICD)-9 codes for deaths in 1996–1999 or ICD-10 codes for deaths in 2000–2010. The cause categories included congenital conditions, immaturity-related conditions, asphyxia, sudden infant death syndrome (SIDS), infections, external causes, other specific conditions, and remaining causes. Causes of stillbirth were not presented since there were no remarkable findings.

### Statistical analysis

The comparisons of birth outcomes in First Nations, Inuit and non-Aboriginal groups were based a composite Aboriginal birth identifier using all the three sources of information (mother tongue, place of residence, IRS membership; “yes” in anyone), while the comparisons of perinatal and infant mortality were based on a composite Aboriginal birth identifier using mother tongue and place of residence (“yes” in either). Because of the poor linkage of stillbirths and infant deaths to IRS members (implausibly low infant mortality: 2.0 per 1000), the IRS membership information was not used in the analyses of perinatal and infant mortality among First Nations.

Preterm, SGA and LGA birth rates were calculated per 100 total births (live births plus stillbirths). Stillbirth and perinatal mortality rates were calculated per 1000 total births. Infant, neonatal and postneonatal mortality rates were calculated per 1000 live births. Crude relative risks (RR) with 95% confidence intervals (CI) were calculated to illustrate the magnitude of the risk disparities. Chi-square test was used to examine the statistical significance of rate differences across study groups. Cochran-Armitage test was used to assess trends in outcome rates over time. Logistic regression was employed to obtain the crude and adjusted odds ratios (OR) for assessing whether the elevated risks could be explained by observed maternal characteristics including age (<20, 20–29, 30–34, ≥35 y), marital status (married, common-law union, single/devoiced/widowed), parity (primiparous, multiparous), education [<11 y, 11 y (high school), 12–13 y (college), 14+ y (university)], and rural vs. urban residence. All data analyses were carried out using SAS, Version 9.2.

## Results

Maternal characteristics (age, education, marital status, parity, rural vs. urban residence) differed significantly between Aboriginal and non-Aboriginal births (**Table 1**). First Nations and Inuit mothers were much younger than non-Aboriginal mothers. The proportion of mothers under 20 years of age was substantially higher for Inuit (22.5%) or First Nations (18.3%) vs. non-Aboriginal (3.4%) mothers. First Nations and Inuit mothers had much lower educational attainment. Over 40% of Inuit and First Nations mothers had completed education less than 11 years (high school in Quebec)—over 4 times higher than non-Aboriginal mothers. The proportion of single, divorced or widowed mothers was over 3 times higher in First Nations (28.7%) or Inuit (34.2%) versus non-Aboriginal (8.8%) mothers. The proportion of primiparous women was lower in First Nations (34.0%) or Inuit (28.5%) vs non-Aboriginal (46.3%) mothers. A much higher percentage of births was from rural areas in First Nations (84.5%) or Inuit (97.5%) vs. non-Aboriginal (22.5%) births.

**Table 1:** Maternal characteristics for First Nations, Inuit and Non-Aboriginal singleton births in the study cohort, Quebec 1996–2010.

		First Nations	Inuit	Non-	P
				Aboriginal	
	**N**	20190	4260	229960	
**Maternal age**	Mean±SD (year)	25.6±6.1	24.6±5.9	28.8±5.2	<0.001
**(%)**	<20	18.3	22.5	3.4	<0.001
	20–34	72.8	71.3	82.4	
	>35	9.0	6.2	14.2	
**Education (y)**	Mean/SD	11.0±3.0	10.1±2.5	13.9±3.1	<0.001
**(%)**	<11 y	42.7	52.1	10.5	<0.001
	11 y (High school)	23.4	26.8	15.2	
	12–13y (College)	14.1	13.5	16.0	
	≥14y (Some university)	19.8	7.6	58.3	
**Marital status**	Married	23.8	15.4	41.1	<0.001
**(%)**	common-law union	47.5	50.5	50.1	
	Single, divoiced or widowed	28.7	34.2	8.8	
**Parity (%)**	Primiparous	34.0	28.5	46.3	<0.001
	Multiparous	68.0	71.5	53.7	
**Rural versus**	Rural	84.5	97.5	22.5	<0.001
**urban (%)**	Urban	15.5	2.5	77.5	

In general, the risks of adverse birth outcomes were much higher in First Nations or Inuit vs. non-Aboriginal births (**Table 2**). Preterm birth rates were 1.72 times higher for Inuit and 1.11 times higher for First Nations relative to non-Aboriginal births during the study period. Compared First Nations vs. non-Aboriginal births, LBW (RR = 0.76) and SGA (RR = 0.45) birth rates were substantially lower, while HBW (RR = 2.27) and LGA (RR = 2.80) birth rates were substantially higher. Perinatal and infant mortality rates were 1.47 and 1.80 times higher for First Nations (10.1 and 7.3 per 1000, respectively), and 2.37 and 4.46 times higher for Inuit (16.3 and 18.1 per 1000, respectively) births as compared to non-Aboriginal (6.9 and 4.1 per 1000, respectively) births. Large relative risk elevations were observed in neonatal mortality among Inuit infants (2.94 times), and in postneonatal mortality among First Nations (3.71 times) or Inuit (8.98 times) infants.

**Table 2:** Birth outcomes, perinatal and infant mortality in First Nations, Inuit and Non-Aboriginal singleton births in the study cohort, Quebec 1996–2010.

	FN	Inuit	Non-Ab	RR (95%CI)		RR (95%CI)	
Outcome	(A)	(B)	(C)	(A) vs. (C)	P	(B) vs. (C)	P
**Total births** *	20190	4260	229960				
**Births, %**							
Preterm (<37 weeks)	7.1	11.0	6.4	1.11 (1.06,1.17)	<0.001	1.72 (1.57,1.87)	<0.001
SGA (<10th)	3.9	5.4	8.7	0.45 (0.42,0.49)	<0.001	0.62 (0.55,0.71)	<0.001
LGA (>90th)	25.7	14.9	9.2	2.80 (2.72,2.87)	<0.001	1.62 (1.50,1.74)	<0.001
LBW (<2500 g)	3.6	6.4	4.7	0.76 (0.71,0.82)	<0.001	1.37 (1.22,1.54)	<0.001
HBW (>4000 g)	23.9	12.6	10.5	2.27 (2.21,2.33)	<0.001	1.20 (1.10,1.30)	<0.001
**Deaths, per 1000**							
Perinatal death	10.1	16.3	6.9	1.47 (1.26,1.73)	<0.001	2.37 (1.87,3.01)	<0.001
Stillbirth	6.7	7.5	3.9	1.73 (1.42,2.10)	<0.001	1.94 (1.36,2.75)	<0.001
Infant death	7.3	18.1	4.1	1.80 (1.49,2.17)	<0.001	4.46 (3.54,5.61)	<0.001
Neonatal death	3.5	8.9	3.0	1.15 (0.88,1.51)	0.291	2.94 (2.13,4.07)	<0.001
Postneonatal death	3.8	9.2	1.0	3.71 (2.81,4.90)	<0.001	8.98 (6.41,12.59)	<0.001
**Cause-specific infant**	**death, per**	**1000**					
Congenital anomalies	1.7	2.8	1.1	1.54 (1.04,2.27)	0.029	2.56 (1.44,4.57)	<0.001
Immaturity-related	0.5	2.6	0.8	0.64 (0.32,1.31)	0.221	3.45 (1.87,6.33)	<0.001
Asphyxia	0.7	2.3	0.5	1.23 (0.67,2.28)	0.504	4.36 (2.29,8.30)	<0.001
SIDS	0.9	4.2	0.2	3.97 (2.24,7.04)	<0.001	18.51 (10.85,31.57)	<0.001
Infections	0.9	1.2	0.1	8.76 (4.60,16.7)	<0.001	11.35 (4.33,29.75)	<0.001
External causes	0.5	1.2	0.1	4.00 (1.83,8.79)	<0.001	9.73 (3.76,25.19)	<0.001
Others	2.2	3.8	1.2	1.78 (1.26,2.52)	<0.001	3.08 (1.86,5.09)	<0.001

SGA = small-for-gestational-age (birth weight <10th percentile); LGA = large-for-gestational-age (>90th percentile); LBW = low birth weight (<2500 g); HBW = highh birth weight (>4000 g); FN = First Nations; Non-Ab = Non-Aboriginal; RR = relative risk; CI = confidence interval

*Birth outcomes are based on a composite Aboriginal birth identifier using three sources of information: mother tongue, residential postal code and municipality name, or Indian Registration System membership; mortality outcomes for First Nations were based on 16700 First Nations births identified by mother tongue or residential postal code and municipality, see Methods for details.

Analyses of cause-specific infant mortality rates revealed significant risk elevations for infant death due to congenital anomalies, SIDS, infections and external causes among First Nations or Inuit vs. non-Aboriginal infants, and due to asphyxia or immaturity-related conditions among Inuit vs. non-Aboriginal infants (**Table 2**). Compared to non-Aboriginal infants, large relative risk elevations were observed for infant mortality due to infections for First Nation (RR = 8.76) or Inuit (RR = 11.35), due to SIDS for First Nations (RR = 3.97) or Inuit (RR = 18.51), and due to injuries/external causes for First Nations (RR = 4.00) or Inuit (RR = 9.73) infants.

The trends in disparities in birth outcomes, perinatal and infant mortality are presented in **Table 3**. Compared to non-Aboriginal births, preterm birth rates were persistently (1.7–1.8 times) higher in Inuit, LGA rates were persistently (2.7–3.0 times) higher in First Nations births. SGA rates were persistently lower for First Nations or Inuit vs. non-Aboriginal births, while SGA rates trended higher for Inuit births (increased from 4.7% to 6.4%) only. The disparities in perinatal mortality comparing First Nations or Inuit vs. non-Aboriginal births fluctuated and showed no apparent trends between 1996–2000 and 2006–2010. Surprisingly, the relative risk disparities in infant mortality comparing Inuit vs. non-Aboriginal infants increased from 1996–2000 to 2006–2010 (from 4.1 to 5.2 times). The First Nations vs. non-Aboriginal relative risk disparities in infant mortality fluctuated, narrowing from 1996–2000 (RR = 2.09) to 2001–2005 (RR = 1.36), but widening in the most recent period (RR = 1.93 in 2006–2010). Increasing relative risk disparities were observed in neonatal mortality comparing Inuit vs. non-Aboriginal infants (from 2.76 to 3.29 times), and in postneonatal mortality comparing Inuit (from 6.97 to 12.33 times) or First Nations (from 3.76 to 4.25 times) to non-Aboriginal infants.

**Table 3:** Trends in birth outcomes, perinatal and infant mortality in First Nations (FN), Inuit and Non-Aboriginal (Non-Ab) births, Quebec 1996–2010.

	FN	Inuit	Non-Ab	RR (95%CI)		RR (95%CI)	
	(A)	(B)	(C)	(A) vs. (C)	P	(B) vs. (C)	P
**Preterm, %**							
1996–2000	6.8	11.6	6.5	1.04 (0.95,1.14)	0.421	1.78 (1.53,2.07)	<0.001
2001–2005	7.3	10.8	6.6	1.11 (1.01,1.22)	0.026	1.65 (1.41,1.92)	<0.001
2006–2010	7.3	10.6	6.2	1.18 (1.09,1.29)	<0.001	1.72 (1.49,2.00)	<0.001
P for trend	0.244	0.394	0.003				
**SGA, %**							
1996–2000	4.2	4.7	9.4	0.45 (0.40,0.51)	<0.001	0.50 (0.39,0.64)	<0.001
2001–2005	4.0	5.0	8.1	0.49 (0.43,0.55)	<0.001	0.61 (0.48,0.77)	<0.001
2006–2010	3.6	6.4	8.5	0.43 (0.38,0.48)	<0.001	0.76 (0.62,0.92)	0.004
P for trend	0.058	0.043	<0.001				
**LGA, %**							
1996–2000	24.4	14.5	9.1	2.67 (2.55,2.81)	<0.001	1.59 (1.39,1.81)	<0.001
2001–2005	26.3	15.8	9.7	2.73 (2.6,2.86)	<0.001	1.64 (1.45,1.86)	<0.001
2006–2010	26.3	14.3	8.8	2.98 (2.85,3.11)	<0.001	1.62 (1.43,1.84)	<0.001
P for trend	0.011	0.922	0.052				
**Perinatal death**	**, per**	**1000**					
1996–2000	10.0	18.4	6.6	1.52 (1.14,2.02)	0.004	2.79 (1.87,4.15)	<0.001
2001–2005	9.2	14.2	6.9	1.33 (0.99,1.78)	0.056	2.05 (1.32,3.20)	0.001
2006–2010	11.0	16.4	7.1	1.56 (1.21,2.00)	<0.001	2.31 (1.56,3.44)	<0.001
P for trend	0.604	0.655	0.253				
**Stillbirth**	**, per**	**1000**					
1996–2000	6.2	10.3	3.7	1.69 (1.18,2.44)	0.004	2.83 (1.66,4.82)	0.001
2001–2005	6.6	5.7	3.8	1.73 (1.22,2.46)	0.002	1.50 (0.74,3.01)	0.258
2006–2010	7.1	6.6	4.1	1.75 (1.28,2.39)	<0.001	1.61 (0.86,3.00)	0.136
P for trend	0.534	0.234	0.165				
**Infant death**	**, per**	**1000**					
1996–2000	9.1	17.9	4.4	2.09 (1.54,2.84)	<0.001	4.10 (2.72,6.18)	<0.001
2001–2005	5.5	16.4	4.0	1.36 (0.93,1.99)	0.110	4.08 (2.68,6.22)	<0.001
2006–2010	7.3	19.8	3.8	1.93 (1.41,2.63)	<0.001	5.19 (3.58,7.52)	<0.001
P for trend	0.266	0.710	0.084				
**Neonatal death**	**, per**	**1000**					
1996–2000	3.9	8.2	3.0	1.31 (0.83,2.07)	0.244	2.76 (1.51,5.05)	<0.001
2001–2005	2.6	8.6	3.1	0.84 (0.49,1.45)	0.537	2.73 (1.53,4.87)	<0.001
2006–2010	3.9	9.9	3.0	1.30 (0.86,1.98)	0.212	3.29 (1.96,5.53)	<0.001
P for trend	0.979	0.632	0.874				
**Postneonatal**	**death, per**	**1000**					
1996–2000	5.3	9.8	1.4	3.76 (2.47,5.73)	<0.001	6.97 (3.93,12.36)	<0.001
2001–2005	2.8	7.9	0.9	3.19 (1.82,5.60)	<0.001	8.90 (4.70,16.83)	<0.001
2006–2010	3.4	10.0	0.8	4.25 (2.61,6.93)	<0.001	12.33 (7.07,21.52)	<0.001
P for trend	0.118	0.958	<0.001				

The crude and adjusted ORs of adverse outcomes comparing First Nations and Inuit vs. non-Aboriginal births are presented in **Table 4**. The adjustments for maternal characteristic (age, education, marital status, parity and rural vs. urban residence) attenuated the risk differences in perinatal, infant and postneonatal mortality comparing First Nations or Inuit vs. non-Aboriginal births. However, significantly elevated risks remained in perinatal, infant and postneonatal mortality comparing First Nations (adjusted ORs: 1.28, 1.47 and 2.28, respectively) or Inuit (adjusted ORs: 1.70, 3.66 and 6.01, respectively) to non-Aboriginal births. Significantly elevated risks of preterm birth (adjusted OR = 1.33) and neonatal mortality (adjusted OR = 2.44) remained for Inuit infants, while the lower risk of SGA and higher risk of LGA for First Nations and Inuit births were even more striking after the adjustments.

**Table 4:** Crude and adjusted odds ratios (ORs) of adverse outcomes comparing First Nations or Inuit vs. Non-Aboriginal births, Quebec 1996–2010.

	First Nations vs Non-Aboriginal				Inuit vs Non-Aboriginal			
	Crude		Adjusted *		Crude		Adjusted *	
	OR (95%CI)	P	OR (95%CI)	P	OR(95%CI)	P	OR(95%CI)	P
**Births**								
Preterm	1.12 (1.06,1.19)	0.001	0.99 (0.93,1.05)	0.722	1.81 (1.64,1.99)	<0.001	1.33 (1.19,1.5)	<0.001
SGA	0.43 (0.4,0.46)	<0.001	0.35 (0.32,0.38)	<0.001	0.60 (0.53,0.69)	<0.001	0.50 (0.43,0.58)	<0.001
LGA	3.42 (3.3,3.54)	<0.001	3.67 (3.52,3.83)	<0.001	1.73 (1.59,1.88)	<0.001	1.79 (1.62,1.97)	<0.001
LBW	0.76 (0.7,0.82)	<0.001	0.60 (0.55,0.66)	<0.001	1.39 (1.23,1.58)	<0.001	0.90 (0.77,1.04)	0.163
HBW	2.67 (2.58,2.77)	<0.001	3.02 (2.9,3.15)	<0.001	1.23 (1.12,1.34)	<0.001	1.38 (1.25,1.53)	<0.001
**Deaths**								
Perinatal death	1.48 (1.26,1.73)	<0.001	1.28 (1.04,1.57)	0.019	2.40 (1.88,3.05)	<0.001	1.70 (1.22,2.36)	0.002
Stillbirth	1.73 (1.42,2.11)	<0.001	1.47 (1.12,1.92)	0.005	1.94 (1.36,2.77)	<0.001	1.03 (0.59,1.82)	0.910
Infant death	1.81 (1.49,2.18)	<0.001	1.47 (1.17,1.84)	0.001	4.52 (3.58,5.71)	<0.001	3.66 (2.77,4.83)	<0.001
Neonatal death	1.16 (0.88,1.51)	0.291	1.07 (0.78,1.47)	0.670	2.96 (2.13,4.11)	<0.001	2.44 (1.63,3.66)	<0.001
Postneonatal death	3.72 (2.82,4.92)	<0.001	2.28 (1.63,3.20)	<0.001	9.06(6.45,12.73)	<0.001	6.01 (4.05,8.90)	<0.001

SGA = small-for-gestational-age (birth weight <10th percentile); LGA = large-for-gestational-age (>90th percentile); LBW = low birth weight (<2500 g); HBW = high birth weight (>4000 g)

* Adjusted for maternal age, marital status, parity and education and rural vs. urban residence.

Similar risk patterns and risk differences were observed if the comparisons of First Nations and Inuit vs. non-Aboriginal birth outcomes, perinatal and infant mortality were restricted to rural areas (data not shown).

**Table 5** presents the outcome rates for Aboriginal groups by different Aboriginal birth identifiers. Preterm birth rates were lower for First Nations births identified by IRS membership (6.6%) than those identified by mother tongue (7.7%) or residential postal code (7.1%). SGA birth rates were identical (3.6%) for First Nations births identified by residential postal code vs. IRS membership, and both were higher than the SGA rate for First Nations births identified by mother tongue (2.8%). LGA birth rates were similar for First Nations births identified by residential postal code / municipality name (27.2%) and IRS membership (26.4%), and both were higher than the rate for First Nations births identified by mother tongue (29.7%). Stillbirth, perinatal, infant, neonatal and postneonatal mortality rates were all higher for First Nations births identified by mother tongue than those First Nations births identified by residential postal code / municipality name; the relative risk differences were in the range of 15–25%. The relative risk differences in adverse birth outcomes were in the range of 1–12% for Inuit births identified by mother tongue vs residential postal code and municipality name.

**Table 5:** Birth outcomes, perinatal and infant mortality for First Nation and Inuit singleton births identified by different methods, Quebec 1996–2010.

	First	Nations	identified	by*:			Inuit	by*	
	Mother	Postal	IRS				Mother	Postal	
	tongue	code	membership	Ratio	Ratio	Ratio	tongue	code	Ratio
	A	B	C	B vs. A	C vs. A	C vs. B	D	E	E vs. D
Total births, n	7020	16150	17790				3560	4180	
**Births, %**									
Preterm	7.7	7.1	6.6	0.93	0.86	0.93	10.2	10.8	1.06
SGA	2.8	3.6	3.6	1.29	1.31	1.01	5.5	5.2	0.95
LGA	29.7	27.2	26.4	0.92	0.89	0.97	14.8	14.9	1.01
LBW	3.3	3.4	3.1	1.04	0.93	0.89	5.7	6.1	1.07
HBW	26.7	25.2	24.7	0.94	0.93	0.98	12.4	12.6	1.02
**Deaths, per 1000**									
Perinatal death	12.4	9.7	NA	0.78	NA	NA	14.9	16.7	1.12
Stillbirth	8.4	6.3	NA	0.75	NA	NA	6.8	7.6	1.12
Infant death	8.9	7.1	NA	0.80	NA	NA	17.0	18.8	1.11
Neonatal death	4.0	3.4	NA	0.85	NA	NA	8.2	9.1	1.11
Postneonatal death	4.9	3.7	NA	0.76	NA	NA	8.9	9.7	1.09

IRS = Indian registration system; SGA = small-for-gestational-age (birth weight <10th percentile); LGA = large-for-gestational-age (>90th percentile); LBW = low birth weight (<2500 g); HBW = high birth weight (>4000 g); NA = not available (due to poor quality of data linkage of stillbirths and infant deaths to IRS members).

* We did not test whether the outcome differences among the three First Nations groups or between the two Inuit groups were statistically significant because these groups are not mutually exclusive or not independent (ex. the same person could be identified as a First Nation by any of the three identifiers, and thus could be counted in each of the three First Nation groups).

## Discussion

### Main findings

We found persistently higher rates of preterm birth in Inuit vs. non-Aboriginal births, and of LGA birth in First Nations vs. non-Aboriginal births, and widening disparities in neonatal mortality and infant mortality rates comparing Inuit vs. non-Aboriginal infants, and in postneonatal mortality rates comparing First Nations or Inuit vs. non-Aboriginal infants over the recent decade (between 1996–2000 and 2006–2010) in Quebec. Maternal characteristics could only partly explain the higher risks of perinatal and infant mortality among First Nations and Inuit populations.

### Comparisons with previous studies

Aboriginal peoples are a potent mirror of human multiplicity of culture, language, and spirit, yet they are frequently marginalized [22], and suffer substantially higher risks of adverse birth outcomes and infant mortality even in developed countries.^1–14^ Our results confirmed the elevated risks of perinatal and infant mortality among First Nations and Inuit in Quebec [3,4,11]. Moreover, infant mortality was 19.8 per 1000 for Inuit infants in Quebec 2006–2010, surprisingly higher than that of 16.1 per 1000 for Aboriginal infants in 1998–2001 in Western Australia–a previously reported worst Aboriginal infant mortality rate in developed countries [12]. More worrisomely, the widening disparities in infant mortality comparing Inuit vs. non-Aboriginal infants, and in postneonatal mortality comparing First Nations or Inuit vs. non-Aboriginal infants suggest worsening infant health inequalities over the recent decade in Quebec, strongly indicating the need for monitoring the trends in other regions in Canada. Such increasing Aboriginal vs. non-Aboriginal disparities in infant mortality have also been reported in Western Australia between 1980–1984 and 1998–2001 (RR increased from 3.0 to 4.4) [12]. The observed large relative risk disparities in postneonatal mortality comparing First Nations or Inuit to non-Aboriginal infants may reflect the impacts of socioeconomic conditions, environmental factors and infant care [23,24]. Aboriginal peoples are often disadvantaged such as lack of political representation, marginalization, poverty, and lack of access to social services.^22^ These disadvantages are likely the root causes of poor perinatal and infant health.

Large risk elevations were observed for infant death due to preventable causes including infections, SIDS and injuries among First Nations and Inuit infants in Quebec. SIDS was a leading cause of infant mortality among both First Nations and Inuit infants in Quebec. Unsafe sleep environment (non-supine sleep position, bed-sharing) has been recognized as a major risk factor of SIDS [25,26]. Effective promotion of safe sleep environment (back-to-sleep, avoidance of bed-sharing) may help to decrease the risk of SIDS. Improvements in living/housing conditions and infant care environment for Aboriginal families may help to reduce the risk of infant death due to infections and external causes. The higher risk of infant mortality due to congenital anomalies in Aboriginal infants is another cause of concern. The high prevalence of alcohol and tobacco abuse among Aboriginal women [27,28] may partly account for the elevated rates of congenital anomalies. Besides, the elevated risk may result from inadequate prenatal screening for congenital anomalies, or more frequently exposed to environmental teratogens (e.g. through the consumption of sea fish enriched with contaminants through the food chain) in Aboriginal populations.

Consistent with the findings from previous studies, we observed substantially higher rates of preterm birth in Inuit, and of LGA birth in First Nations and Inuit vs. non-Aboriginal births [3,11]. In contrast to the higher risk of SGA in Australian aboriginal vs. non-Aboriginal infants [29], a lower risk of SGA was observed among Inuit and First Nations infants in Quebec, suggesting that poor fetal growth is not a cause of their higher perinatal and infant mortality rates.

### Strengths and limitations

The main strengths are the large population-based birth cohort, and the use of multiple sources of information to identify Aboriginal births allowing more complete identification of Aboriginal births than previous studies. However, misclassifications may occur as births to Aboriginal women (First Nations, Inuit or Metis) who neither reported an Aboriginal mother tongue, nor resided in an Aboriginal community or registered as a First Nation would have been classified as “non-Aboriginal” births. Nevertheless, such misclassifications are likely a very small number of the “non-Aboriginal group”, and would only tend to deflate the observed risk disparities. Similarly, some births to non-Aboriginal women in Aboriginal communities might have been misclassified as “Aboriginal”—this would also attenuate the observed risk differences. Also, we had limited information on maternal characteristics, with no information on many risk factors of adverse birth outcomes such as smoking, alcohol and substance use. However, most of these factors may be considered as mediating risk factors for the poorer outcomes in Aboriginal populations, and thus should not be adjusted for in quantifying the risk disparities.

## Conclusions

The persistent and even widening disparities in perinatal and infant health indicators comparing First Nations and Inuit to non-Aboriginal births in Quebec over the recent decade strongly call for effective intervention programs to improve Aboriginal perinatal and infant health, and the need for timely monitoring the trends in other regions in Canada. Possible interventions may include governmental investments for improving socioeconomic conditions and accessibility to high-quality perinatal and infant health care, along with promotion of safe motherhood and infant care for Aboriginal parents and communities. Concerted efforts from all levels of government policy makers, Aboriginal authority and health organizations are needed to make a difference [30].
